# Angiotensin converting enzyme inhibitors do not increase the risk of poor outcomes in COVID-19 disease. A multi-centre observational study

**DOI:** 10.1177/0036933020951926

**Published:** 2020-09-01

**Authors:** Khurram Shahzad Khan, Hamish Reed-Embleton, Jen Lewis, Pamela Bain, Sajid Mahmud

**Affiliations:** 1 ST6 in General Surgery, Department of Surgery, University Hospital Hairmyres, UK; 2 FY1 in General Medicine, Department of Medicine, University Hospital Hairmyres, UK; 3 Research Associate / Medical Statistician, Medical Statistics/Design, Trials & Statistics, School of Health and Related Research, University of Sheffield, UK; 4 Clinical Fellow General Surgery, Department of Surgery, University Hospital Hairmyres, UK; 5 Consultant Surgeon, Department of Surgery, University Hospital Hairmyres, UK

**Keywords:** Hypertension, angiotensin converting enzyme (ACE) inhibitor, mortality, SARS-CoV-2, Scotland

## Abstract

**Background and aims:**

Hypertension is associated with an increased risk of severe outcomes with COVID-19 disease. Angiotensin Converting Enzyme (ACE) inhibitors are widely used as a first line medication for the treatment of hypertension in the UK, although their use was suggested in early reports to increase the risk associated with SARS-CoV-2 infection.

**Methods:**

A prospective cohort study of hospitalised patients with laboratory confirmed COVID-19 was conducted across three hospital sites with patients identified on the 9th April 2020. Demographic and other baseline data were extracted from electronic case records, and patients grouped depending on ACE inhibitor usage or not. The 60-day all-cause mortality and need for intubation compared.

**Results:**

Of the 173 patients identified, 88 (50.8%) had hypertension. Of these 27 (30.7%) used ACE inhibitors. We did not find significant differences in 60-day all-cause mortality, the requirement for invasive ventilation or length of stay between our patient cohorts after adjusting for covariates.

**Conclusion:**

This study contributes to the growing evidence supporting the continued use of ACE inhibitors in COVID-19 disease, although adequately powered randomised controlled trials will be needed to confirm effects.

## Introduction

The SARS-CoV-2 pandemic has resulted in an unprecedented challenge for global healthcare systems. In Scotland there has been over 15,000 cases with over 2,400 deaths by 10/06/2020.^1^ Multiple risk factors for poor outcomes have been identified, most consistently increasing age and underlying health conditions.^2^ Large scale observational studies looking at the characteristics in hospitalised patients in the UK,^2^ Italy,^3^ the USA^4^ and China^5^ have consistently reported chronic cardiac disease or hypertension as the most common comorbidity associated with infection.

In Scotland approximately 30% of adults have hypertension, although the rates in the 2 least deprived quintiles is only around 24%.^6^ The National Institute of Health and Care Excellence (NICE) guidelines in the UK recommend Angiotensin Converting Enzyme (ACE) inhibitors as one of the first line therapies for the management of patients with hypertension.^7^ ACE is a functional part of the Renin-Angiotensin system (RAS) whose primary roles include vasoconstriction, raising blood pressure, promotion of inflammation, and fibrosis.^8^ ACE2 receptors, which are used by coronaviruses to gain entry to target cells, antagonise these effects and are found in the heart, kidneys, lung alveolar type II epithelial cells and testes.^8^ Early reports suggested that ACE inhibitors may lead to a compensatory upregulation of ACE2, and that the continued use of these medications may have deleterious effects on patients with COVID-19 disease.^9^

Currently, the European Society of Cardiology consider that ACE inhibitors are safe and support their continued usage according to national guidelines, and note the lack of data supporting harmful effect.^10^ In the UK, NICE also cite the poor quality of available studies and were unable to conclude whether ACE inhibitors increase the risk of infection or severe outcomes with SARS-CoV-2 infection.^11^

We aim to explore the potential influences of ACE inhibitors on a hospitalised patient population with hypertension and COVID-19 disease and determine whether continued usage may negatively impact patient outcomes.

## Material and methods

This was a prospective cohort study carried out on all hospitalized patients with real-time transcriptase polymerase chain reaction (RT-PCR) confirmed COVID-19, on a single day (Friday the 9th April 2020). Patients from three acute hospitals in a single National Health Service (NHS) Trust in Scotland serving a population of 658,130 with over 1,660 in-patient’s bed capacity were included. COVID-19 positive patients were identified from TrakCare Electronic Medical Record System. The RT-PCR swabs were carried out on symptomatic patients or patients suspected of having COVID-19. Patients with an initial negative swab who later during their hospital admission had a positive swab were also included in the study. The electronic case notes were analysed for baseline characteristics; age, gender, co-morbidities and admission blood test.

Scottish Index of Multiple Deprivation (SIMD)^12^ score and Age-Adjusted Charlson Comorbidity Index (ACCI)^13^ score were analyzed. SIMD utilises postcode to calculate a score of relative deprivation taking into account income, health, education crime, housing, employment and access to services. The ACCI is a score that predicts 10 year mortality, it includes 19 medical conditions, and provides an age adjusted score for every year above 50 years of age. Patients with the past medical history of hypertension were included and those without hypertension excluded. Patients were divided into two groups based on if they were receiving ACE inhibitors or not.

Critical care was defined as care provided above general ward level. Mortality was in-patient all-cause mortality. All patients were followed up for at least 60 days from admission or until discharge or death. Poor outcome was defined as either need for intubation and/or death. This was to account for those patients who were critically ill but not suitable for intubation and were Do Not Attempt Resuscitation and/or palliated. Patients transferred to off-site rehabilitation unit were considered to be discharged.

### Statistical analyses

Univariate analyses were carried out to explore whether differences existed in outcomes between hypertensive patients with and without treatment with ACE inhibitors. Subsequently, multivariate analyses were performed to examine whether any differences between groups remained after controlling for covariates. A logistic regression was performed to explore whether treatment with ACE inhibitors increase the risk of intubation and ventilation. A multivariate linear regression was used to explore effects on length of stay, and a multivariate Cox’s proportional hazards model was used to explore the effects of ACE inhibitors on survival.

This study was registered with the NHS Lanarkshire’s Clinical Quality Project, project id: 13139. As this was an observational study, patient consent was not required.

## Results

**Figure 1.**
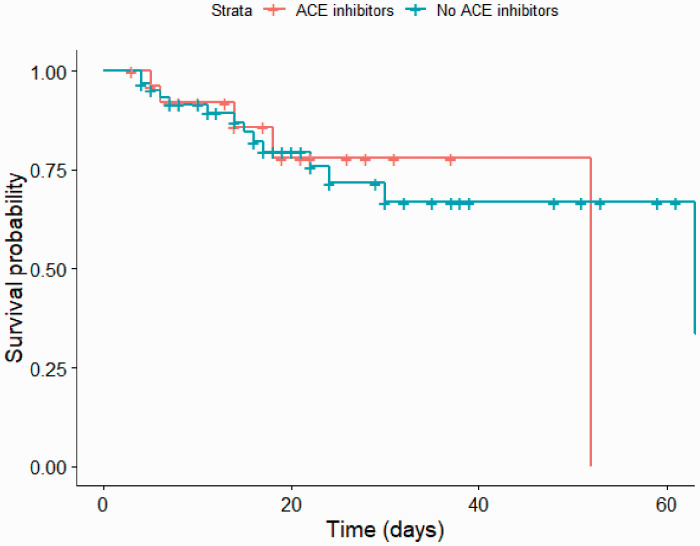
Unadjusted Kaplan-Meier plot comparing inpatients using ACE inhibitor and those who were not. Crosses on this plot represent patient discharges.

There were 173 patients identified with positive RT-PCR, of these 88 (50.8%) had hypertension. Of the hypertensive patients 50 (56.8%) were male, with a mean age of 72 (±13.5) years. Summary demographics and baseline investigation results were calculated, and differences explored with univariate analyses (Table 1). A total of 19 deaths occurred in 60 days. We did not find significant differences in the outcomes between the cohorts using ACE inhibitors and those who were not (Table 2). Figure 1 represents an unadjusted Kaplan-Meier plot comparing survival probabilities in those using ACE inhibitors and those who are not.

**Table 1.** Demographics and baseline investigation results.

	All participants(n = 88)	No ACE inhibitors(n = 61)	ACE inhibitors(n = 27)	Difference^a^	95% CI	p
Mean age (SD)	72.03 (13.51)	74.61 (13.33)	66.22 (12.25)	9.00	3, 15	0.006^b^
N Male (%)	50 (56.82)	30 (49.18)	20 (74.07)	0.22	0.01, 0.42	0.052
Median ACCI (IQR)	4.5 (3, 6)	5 (4, 7)	3 (2, 5)	2.00	1–3	0.002^b^
Median^c^ deprivation decile (IQR)	4 (2, 6)	4 (2, 6)	4 (2.5, 7)	–1.00	–2, 0	0.247
Median Cr (IQR)	93 (70.8, 115.2)	94 (67, 115)	91 (76.5, 115)	–1.00	–17, 15	0.968
Median CRP (IQR)	57.5 (31.8, 132)	52 (28, 124)	85 (46.5, 144.5)	–19.00	–49, 8	0.182
Median lymphocytes (IQR)	0.85 (0.7, 1.2)	0.9 (0.7, 1.3)	0.8 (0.7, 1.1)	<0.01	–0.2, 0.2	0.841
Median neutrophils (IQR)	5.95 (4, 8)	5.6 (3.9, 7.6)	6.4 (4.2, 9.2)	–0.60	–2, 0.9	0.455
Median platelets (IQR)	205.5 (161.2, 257.5)	208 (171, 255)	182 (151.5, 258)	11.00	–28, 52	0.566
N eGFR >59 (%)	48 (54.55)	31 (50.82)	17 (62.96)	0.10	–0.11, 0.32	0.411
N CXR > 1 (%)^c^	40 (45.45)	25 (40.98)	15 (55.56)	0.11	–0.11, 0.33	0.366

ACE: angiotensin converting enzyme; SD: standard deviation; ACCI: age-adjusted Charlson Comorbidity Index; IQR: interquartile range; Cr: creatinine; CRP: C-reactive protein; eGFR: estimated glomerular filtration rate; CXR: Chest X-ray.

**^a^**Difference column shows location difference estimate for numeric variables, proportion difference estimate for categorical variables

^b^Significant at p<0.01.

‡Variables had missing values (Dep. Decile = 1, CXR = 2).

**Table 2.** Comparison of outcomes.

	All participants(n = 88)	No ACE inhibitors (n = 61)	ACE inhibitors(n = 27)	Difference^a^	95% CI	p
Critical care admission (%)	18 (20.45)	9 (14.75)	9 (33.33)	0.24	–0.04, 0.53	0.088
Intubated and ventilated (%)	12 (13.64)	7 (11.48)	5 (18.52)	0.13	–0.22, 0.47	0.582
In-patient mortality (%)^b^	19 (21.59)	14 (22.95)	5 (18.52)	–0.06	–0.32, 0.20	0.853
Median LOS (IQR)^b^	17 (8, 24)	17 (10, 29)	14 (8, 22)	2.00	–3.00, 8.00	0.337

ACE: angiotensin converting enzyme; LOS: length of stay; IQR: interquartile range.

^a^Difference column shows location difference estimate for numeric variables, proportion difference estimate for categorical variables.

^b^Variables missing data from remaining in-patients (n=2).

Treatment with ACE inhibitors remained insignificant in predicting invasive ventilation when controlling for ACCI and sex. Of the included covariates, only ACCI was significant (OR = 0.551, p < 0.001) suggesting that patients with a higher ACCI were less likely to be admitted to ICU. Treatment with ACE inhibitors remained insignificant on 60-day all-cause mortality after adjusting for sex, ICU admission, and age adjusted ACCI. Treatment with ACE inhibitors again remained insignificant for predicting LOS when controlling for covariates, although ACCI (p = 0.012), and admission to ICU (p = 0.014) were significant predictors of long LOS.

## Discussion

This study provides a comprehensive assessment of the influence of ACE inhibitors on poor COVID-19 disease outcomes in a hospitalised population. We did not find significant differences in mortality, the requirement for invasive ventilation or length of stay after adjusting for covariates including sex and age-adjusted Charlson comorbidity index scores. This data contributes to the evidence suggesting the safe continued use of ACE inhibitors in patients as per national guidelines for the management of hypertension.

Early reports on the use of ACE inhibitors were inconsistent with studies suggesting they could be beneficial, harmful or have no effect in COVID-19 disease.^14–16^ Sommerstein et al., postulated that ACE inhibitors may have a biphasic effect on patients with those taking this medication being more at risk of infection, but then more likely to have less severe disease.^17^ Our data give no good evidence of significant differences, but were under-powered to determine small differences.

Despite SARS-CoV-2 tropism for the pulmonary parenchyma, in the most severe cases multiorgan failure develops.^18^ The vasodilatory, anti-inflammatory, anti-proliferative and antifibrotic effects of ACE2 receptors may in fact, partially counterbalance the deleterious systemic effects often observed in COVID-19 disease.^19^,^20^ In experimental conditions, the administration of ACE inhibitors upregulated the activity of ACE2 receptors.^21^ It is now thought that ACE inhibitors may exert a protective effect against the development of acute lung injury in infections with SARS coronaviruses, by limiting the dysregulation of mechanisms that lead to acute respiratory distress and endothelial damage.^22^,^23^

SARS-CoV-2 appears to have a 10-20 fold greater affinity for ACE2 receptors, compared with the SARS-CoV virus which caused the 2003 SARS outbreak, which is suggested as one of the contributing factors for its effective human-to-human transmission.^24^ In propensity score-matched analyses in an outpatient population with positive SARS-CoV-2 infection, ACE inhibitors resulted in decreased hospital admissions and did not confer any added risk of mortality to inpatients.^23^ Our study is unable to determine the potential increased risk of infection in those who were using ACE inhibitors in the community as we only collected data in hospitalised patients. Inhibitors of the RAS system do not play a direct antiviral role, but act indirectly though regulation of the immune system and inflammatory responses.^25^

Our study adds to other studies which support the safe continuation of ACE inhibitors during the current pandemic.^18^,^23^,^25–28^ Further adequately powered trials are required to determine any protective effect of ACE inhibitors in patients with hypertension.

Our cross-sectional methods of identifying all sequential admissions during a defined time period enables a valid representation of the hospital population in Scotland and data was collected across all departments, but we acknowledge that generalisation of our findings is limited by small sample size. Although data was collected across three hospital sites, they were all based in a single NHS health board and this method of data collection will also be subject to a degree of length-time bias.^29^ Recruiting patients from hospitals, care homes and the community as well as reaching a greater sample size with a control population, would improve the validity and generalisability of the data. When examining mortality, Schoenfield tests revealed deviations from proportionality for ACCI (p < 0.05). However, stratification on ACCI was not possible due to a low number of deaths among those with low ACCI scores. Results are therefore reported for the full model, but these should be interpreted with caution.

## Conclusion

We did not find any negative effects on severe outcomes in our hypertensive patient cohort using ACE inhibitors compared with those who did not. Adequately powered trials are needed to determine the optimal treatment of hypertension during the COVID pandemic.
